# Spinach (*Spinacia oleracea*) as green manure modifies the soil nutrients and microbiota structure for enhanced pepper productivity

**DOI:** 10.1038/s41598-023-31204-8

**Published:** 2023-03-13

**Authors:** Ryeong-Hui Kim, Setu Bazie Tagele, Minsoo Jeong, Da-Ryung Jung, Dokyung Lee, TaeHyung Park, Bashizi Flory Tino, Kyeongmo Lim, Min A. Kim, Yeong-Jun Park, Jae-Ho Shin

**Affiliations:** 1 Department of Integrative Biology, Kyungpook National University, Daegu, 41566 Republic of Korea; 2 Department of Applied Biosciences, Kyungpook National University, Daegu, 41566 Republic of Korea; 3 NGS Core Facility, Kyungpook National University, Daegu, 41566 Republic of Korea

**Keywords:** Microbial communities, Microbial ecology, Field trials

## Abstract

Spinach has been suggested as a potential rotation crop for increasing crop yield by enhancing beneficial fungal microbes in continuous monocropping. However, no research on the use of spinach as a green manure has been reported. Thus, we tested the effects of spinach and Korean mustard cultivars (green and red mustards) (10 g pot ^−1^) as green manure on soil chemical properties, pepper productivity, and soil microbiome of long-year pepper-monocropped soil. Spinach improved the soil nutrition (e.g., pH, SOM, TN, NH_4_^+^, and K), weed suppression, and pepper growth. Spinach had by far the highest fruit yield, over 100% pepper fruit yield increment over the mustard green manures and control. Our study showed that the major influencing factors to cause a shift in both bacterial and fungal community assemblies were soil pH, TC TN, and K. Following green manure amendment Bacillota, especially *Clostridium, Bacillus* and *Sedimentibacter,* were enriched, whereas Chloroflexi and Acidobacteriota were reduced. In addition, spinach highly reduced the abundance of Leotiomycetes and *Fusarium* but enriched *Papiliotrema*. FAPROTAX and FUNGuild analysis revealed that predicted functional profiles of bacterial and fungal communities in spinach-amended soil were changed. Spinach-treated soil was differentially abundant in function related to hydrocarbon degradation and functional guilds of symbiotrophs and ectomycorrhizal. This study contributes significantly to our understanding of how the soil fertility and soil microbiome alteration via spinach green manure application as a pre-plant soil treatment might help alleviate continuous cropping obstacles.

## Introduction

Continuous monocropping is a modern agricultural practice in many parts of the world to increase yield on limited land^1–3^. To achieve and maintain high yields and economic benefits, high-value crop production is managed intensively year-round by applying high input chemical fertilizers and agricultural pesticides^2,3^. However, as a result of these practices, the deterioration of soil quality year after year as a result of soil acidification and imbalance of soil nutrient and the microbiome^4^, which ultimately affect plant growth and causes continuous cultivation obstacles, has raised concerns about the sustainability of agroecosystems^5,6^. Similarly, soil nutrient imbalance and soil contamination are serious soil threats in South Korea, for which the government has devised action plans for sustainable soil management^7^. Several measures have been proposed to overcome continuous cultivation obstacles, including crop rotation, chemical fumigation, soil solarization, and organic amendment^8–10^.

Green manuring is an eco-friendly agricultural practice that improves soil fertility and crop productivity while alleviating impediments to continuing cultivation^1,11^. The use of green manures is widely practiced as a sustainable agricultural soil management option because it improves the biological, physical and chemical properties of soil^11–13^. Green manures scavenge nutrients from the soil, prevent nutrient leaching, and slowly release the nutrients that they have absorbed and locked in during decomposition^14^. Incorporating green manures into the soil increases organic matter in the soil, which improves soil structure and fertility, allowing for better plant growth^15^. In addition, many beneficial microbes that play a major role in soil nutrient cycling, soil health, and crop productivity have been found to be stimulated by incorporating green manures into the soil^13,16,17^. The modification of soil nutrients, which ultimately alter soil microbe growth and colonization is partly responsible for the change in soil microbial community structure following addition of green manure^18–20^. For instance, after green manuring, a nutrient-rich environment favors copiotrophs^4,21,22^. while a similar environment discourages slow-growing oligotrophic bacteria^23^. The strong positive link between the alteration of soil microbial community and suppression of soil-borne pathogens^24^. suggests that green manure not only improves soil nutrition but also enriches beneficial microbes with bio-control potential^17^. However, depending on the type of green manure utilized, the efficacy of green manuring might vary greatly^25^.

Spinach, a cool-season vegetable that matures quickly, has been suggested as a potential rotation crop for increasing cucumber yield by enhancing beneficial fungal microbes in continuous monocropping^26^. Nevertheless, to our knowledge, there has not been any recorded research on the usage of spinach as a green manure. Furthermore, *Brassica* species, when used as a green manure, also contain glucosinolates (GSLs), which are hydrolyzed into isothiocyanates and become toxic to soil-borne pests and weeds. Thus, GSL-containing brassicas, such as mustard cultivars, would have better effects on green manuring to effectively alleviate the continuous cultivation obstacles^27^. Nevertheless, the effect of green manuring with Korean mustard cultivars (green and red mustard) and spinach on the productivity of chili pepper (*Capsicum annum*) and the taxonomic and functional diversity of the soil bacterial and fungal communities remains unknown. Chili pepper is a highly profitable crop grown in many parts of the world, including South Korea. However, a recent study found that long-term pepper monoculture made the soil more acidic, causing a significant effect on soil microbial communities^28^. Given that spinach matures quickly, grows in autumn and spring when pepper cultivation does not overlap, and is also nutrient-rich with the potential to increase soil suppressiveness and plant productivity through soil microbiota modification^26^, we hypothesize that spinach would be a suitable alternative to other green manures for tackling soil sickness brought on by long-term monocropping. Thus, we aimed to the investigate the effects of spinach and Korean mustard cultivars as green manures on soil chemical properties, weed suppression, pepper productivity, and soil microbiome.

## Results

### Effect of green manures on soil chemical properties, weed emergence and pepper performance

The impact of green manures on soil chemical properties is shown in Table 1. Although the soil in all treatments was initially taken from a single composite soil sample, the addition of green manures significantly (*p* ≤ 0.05) increased soil pH, NH_4_^+^, and K, but not AP compared to the non-amended control. Spinach also strongly increased EC, SOM, and TN content when compared to control. The highest NO_3_^−^ and K contents were recorded in green mustard- and spinach-amended soils, which were 1.95- and 2.8-times higher than those in control, respectively. Furthermore, green mustard showed the highest C:N ratio while spinach had the lowest. The highest Overall, the nutritional status of the soil was improved by green manures.

**Table 1:** Effect of different green manures on soil chemical properties.

Soil chemical properties	Treatments			
	Control	Spinach	Red mustard	Green mustard
pH	6.17 ± 0.03^c^	6.67 ± 0.03^a^	6.43 ± 0.03^b^	6.47 ± 0.03^b^
EC (dS m^−1^)	1.02 ± 0.00^b^	1.29 ± 0.13^a^	0.91 ± 0.02^b^	0.85 ± 0.01^b^
CEC (cmol_c_ kg^−1^)	17.50 ± 0.10^a^	17.34 ± 0.16^a^	18.15 ± 0.52^a^	17.23 ± 0.32^a^
SOM (g kg^−1^)	26.23 ± 0.24^b^	28.56 ± 0.45^a^	28.05 ± 0.77^a^	24.97 ± 0.53^b^
TN (g kg^−1^)	1.60 ± 0.01^b^	1.90 ± 0.00^a^	1.70 ± 0.00^b^	1.60 ± 0.00^b^
TC (g kg^−1^)	14.66 ± 0.25^c^	14.83 ± 0.39^c^	15.90 ± 0.06^b^	17.00 ± 0.27^a^
C:N ratio	9.18 ± 0.19^b^	7.94 ± 0.09^c^	9.55 ± 0.20^b^	10.63 ± 0.17^a^
NO_3_^−^ (mg kg^−1^)	6.40 ± 1.95^b^	7.73 ± 1.35^b^	9.20 ± 0.25^ab^	12.50 ± 0.32^a^
NH_4_^+^ (mg kg^−1^)	7.43 ± 0.23^c^	8.63 ± 0.20^a^	8.07 ± 0.09^b^	8.17 ± 0.09^ab^
AP (mg kg^−1^)	687.30 ± 2.91^a^	534.49 ± 2.07^d^	582.04 ± 2.55^b^	544.47 ± 2.36^c^
K (cmol_c_ kg^−1^)	0.51 ± 0.01^c^	1.43 ± 0.02^a^	1.01 ± 0.01^b^	0.98 ± 0.01^b^

Control: without green manure amendment.

*EC* Electrical conductivity, *CEC* cation exchange capacity, *SOM* soil organic matter, *TN* total nitrogen, *TC* total carbon, NO_3_^−^ nitrate nitrogen, *NH*_4_^+^ ammonium nitrogen, *AP* available P_2_O_5_, *K* Exchangeable potassium.

Mean values (n = 3) followed by different letter (s) in a row represent significant differences at *p* ≤ 0.05, DMRT test.

The addition of green manures had a remarkable effect on weed emergence reduction and pepper productivity (Table 2). Spinach and green mustard incorporation showed a significant reduction in the emergence of weed populations, particularly monocots, compared with control. In addition, similar to the results of soil nutritional status, control showed the lowest pepper fruit yield whereas spinach had by far the highest fruit yield, over 100% yield increment over control and mustard cultivars. Similarly, spinach improved pepper growth, including plant height, stem diameter, chlorophyll content, canopy diameter, and primary branch diameter. Green mustard also significantly (*p* ≤ 0.05) increased pepper growth compared to control, but showed insignificant (*p* > 0.05) differences in terms of fruit yield.

**Table 2:** Effect of different green manures on weed emergence and pepper performance^a^.

Treatment	Weed emergence (number/pot)			Plant height (cm)	Stem diameter (cm)	Chlorophyll contents (SPAD)	Canopy diameter (cm)	Primary branch length (cm)	Primary branch diameter (cm)	Fruit yield (g plant^−1^)
	Monocot	Dicot	Total							
Control	15.5 ± 1.4^a^	15.8 ± 2.6^a^	31.3 ± 4.9^a^	527 ± 26.7^b^	0.47 ± 0.02^c^	41 ± 0.8^c^	268 ± 23^b^	5.9 ± 1.1^a^	0.11 ± 0.02^c^	30.3 ± 4.0^b^
Spinach	1.5 ± 0.6^c^	2.8 ± 0.8^c^	4.6 ± 1.7^c^	711 ± 21.8^a^	0.58 ± 0.01^a^	52 ± 1.0^a^	418 ± 48^a^	8.2 ± 0.12^a^	0.25 ± 0.02^a^	78.5 ± 1.8^a^
Red mustard	7.2 ± 1.0^b^	10.9 ± 1.3^ab^	18.3 ± 0.8^b^	590 ± 1.7^b^	0.50 ± 0.02^bc^	44 ± 0.2^bc^	299 ± 4^b^	8.2 ± 0.03^a^	0.14 ± 0.01^bc^	39.4 ± 2.7^b^
Green mustard	3.3 ± 0.8^c^	8.7 ± 1.3^b^	12.1 ± 0.6^bc^	582 ± 35.8^a^	0.55 ± 0.01^ab^	45 ± 1.7^b^	290 ± 24^b^	8.3 ± 0.8^a^	0.19 ± 0.01^b^	38.9 ± 2.4^b^

^a^Weed emergence was collected before pepper transplanting, pepper growth traits were determined at the end of the experiment while fruit yield represents three fruit pickings periods.

Mean values followed by different letter (s) in a column represent significant differences at *p* ≤ 0.05, DMRT test.

### Changes in soil microbial diversity and composition structure after green manuring

**Figure 1:**
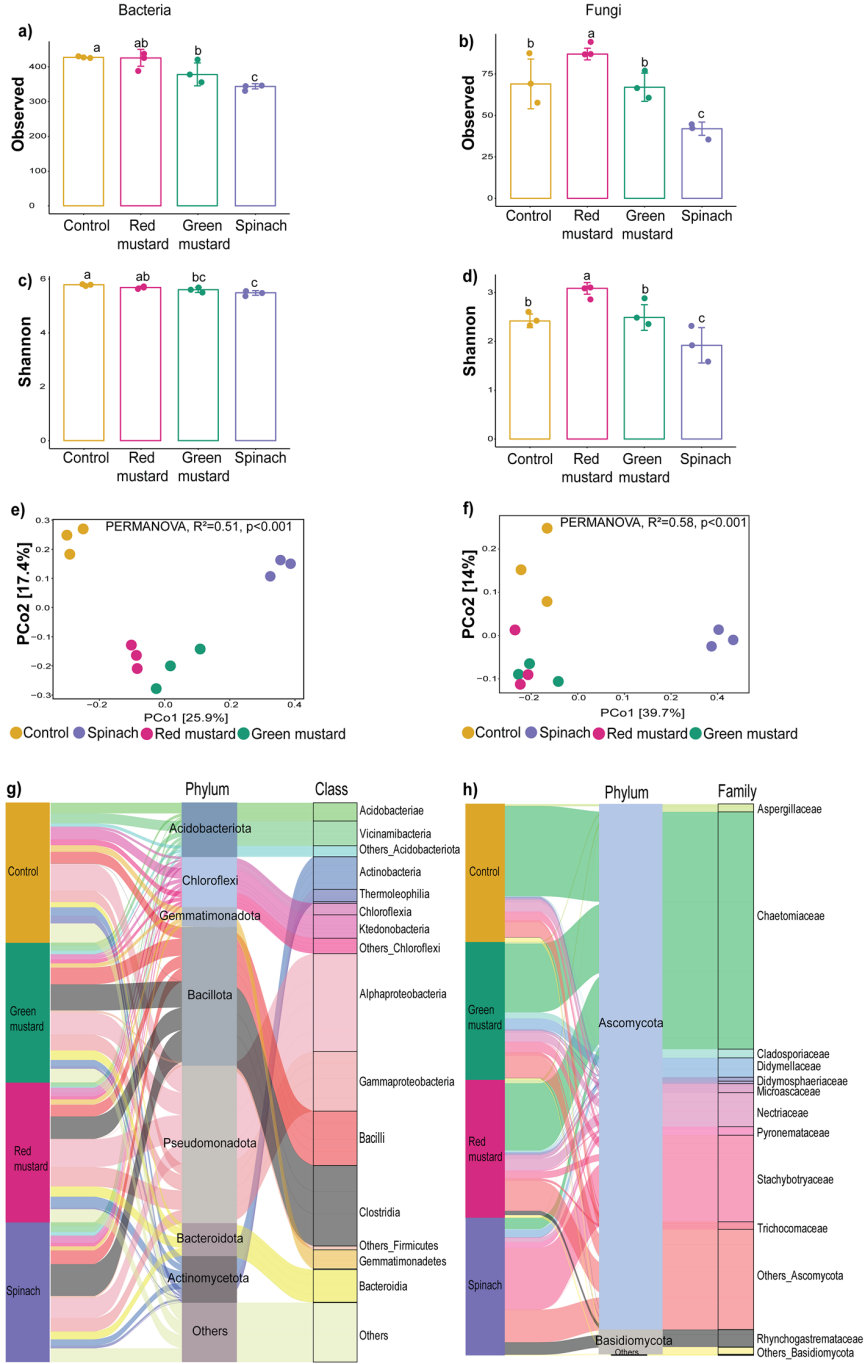
Changes in soil microbial diversity and community structure after green manuring. Alpha diversity of bacteria (**a**,**c**) and fungi (**b**,**d**) in green manure treatments measured by observed and Shannon indices. Mean values (n = 3) followed by different letter (s) in each parameter represent significant differences at *p* ≤ 0.05, DMRT test. Principal coordinate analysis (PCo1 and PCo2) of bacteria (**e**) and fungi (**f**) communities based on Bray–Curtis distance. Taxonomic composition (> 0.1%) of bacterial (**g**) and fungal communities (**h**).

Alpha diversity indices of bacterial and fungal communities were estimated for all green manure amendments, as indicated in Fig. 1a–d and Table S1. Most diversity indices showed that green manuring had a strong negative effect on bacterial diversity than on fungal diversity. Red mustard significantly (*p* ≤ 0.05) increased fungal diversity compared to control and other treatments. Spinach containing no GSL, however, remarkably reduced microbial diversity, implying that the impact of green manuring on soil microbial diversity is highly dependent not only on the type of GSL content but also on other nutritional aspects of the type of green manures.

Green manures had a remarkable impact on the taxonomic composition and structure of bacterial and fungal communities (Fig. 1e,f, Table S2). In comparison to control, all green manures significantly (*p* ≤ 0.05) enriched Bacillota population, while Acidobacteriota and Chloroflexi abundances were greatly reduced (Fig. 1g, Table S2). Bacteroidota abundance was also slightly elevated with green manure amendments. At the class level, Clostridia was the dominant class at all green manure-amended soils, whereas, in control, it was rare group. Spinach also highly reduced the relative abundance of Acidobacteriae (Fig. 1g). Ascomycota was the dominant phylum in the fungal community, accounting for more than 90% of the total across all treatments (Fig. 1h, Table S2). With the exception of spinach, fungal family Chaetomiaceae dominated the phylum Ascomycota. On the other hand, Stachybotryaceae was the most abundant family in spinach-amended soil. Among the other phyla, Basidiomycota, particularly Rhynchogastremataceae, increased in relative abundance with spinach application (Fig. 1h).

### Differential abundant taxa after green manuring

**Figure 2:**
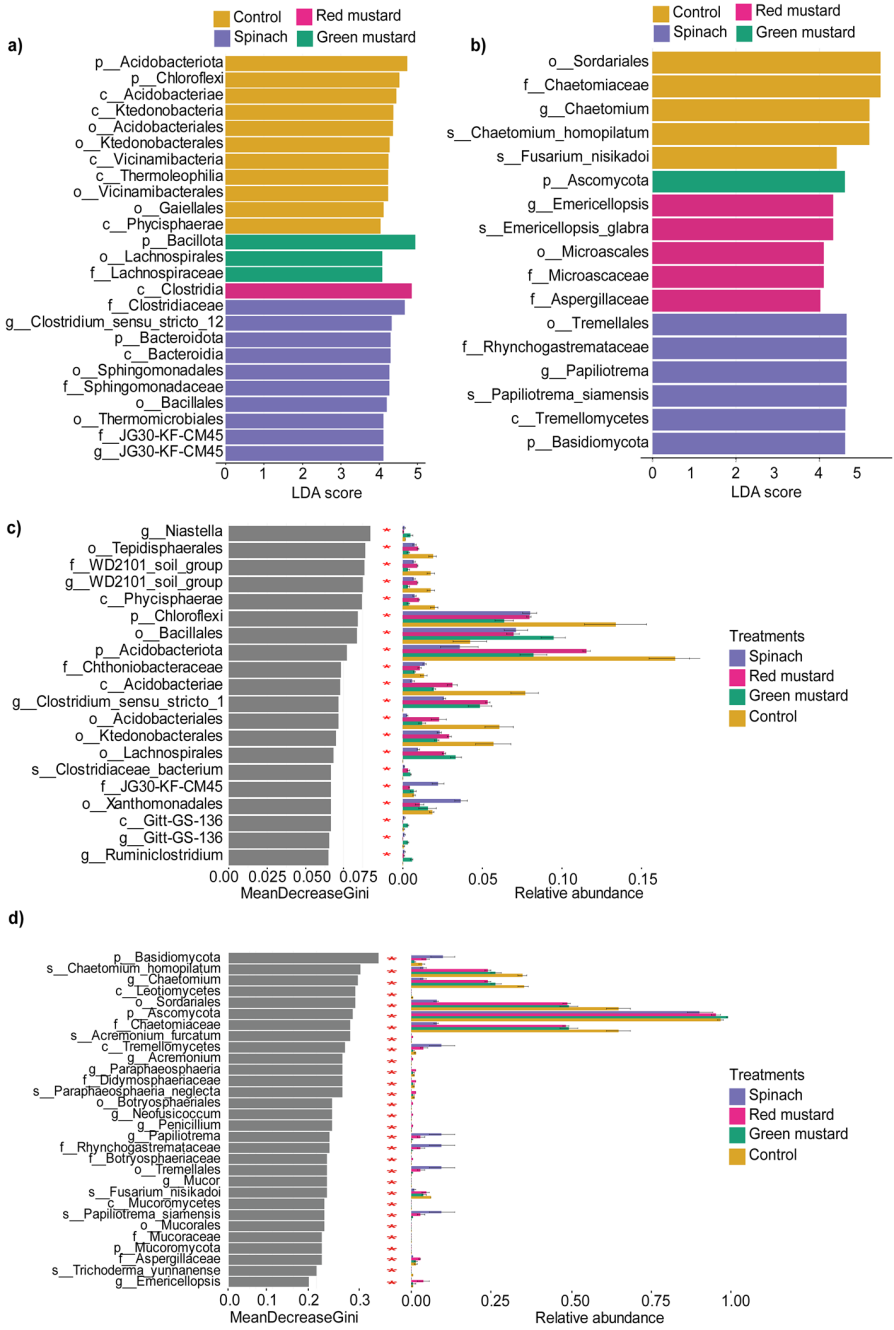
Analysis of differentially abundant taxa after green manuring. Linear discriminatory analysis Effect size (LEfSe) analysis of differentially abundant bacterial (**a**) and fungal (**b**) taxa among the green manures. Random forest graph displaying the most predictive bacterial (**c**) and fungal (**d**) taxa indicators after green manuring. Taxon names are abbreviated as p: phylum, c: class, o: order, f: family, g: genus. s: species.

Potential microbial biomarkers following green manuring were identified using four differential abundance testing tools, namely metastat, metagenomeSeq, LEfSe analysis, and the random forest model (Fig. 2, Supplementary file 2). We identified 160 bacterial and 35 fungal taxa as the key taxa that were differentially abundant between the treatment groups. Significant number of members from p_Acidobacteriota and p_Chloroflexi, such as c_Ktedonobacteria and o_Gaiellales, were reduced in green manured soil compared to control. On the other hand, members of Bacillota including *Clostridium* and *Bacillus* were the most highly stimulated genera in green manure-amended soils and were consistently detected by all the microbiome differential abundance methods (Fig. 2a,c, Supplementary file 2). In addition, several members of f__Sphingomonadaceae, and o__Xanthomonadales, such as *Luteimonas* and *Sphingomonas* were considerably more abundant in spinach-treated soil when compared to control and the other mustard green manures. On the other hand, *Sedimentibacter* was found especially abundant only in mustard-amended soils.

In the fungal community, most tools used in the current study indicated members of Rhynchogastremataceae, such as *Papiliotrema* were the most markedly enriched fungal genera in spinach whereas *Chaetomium* and *Fusarium* were most reduced by the same treatment (Fig. 2, Supplementary file 2). This implies that the stated genera can be considered key fungal biomarkers for spinach. The relative abundance of f_Aspergillaceae and *Emericellopsis* were enriched in red mustard amendment. Furthermore, most biomarker detection tools identified *Chaetomium*, *Fusarium*, and c_Leotiomycetes as the differentially abundant taxa in control (Fig. 2b,d).

### Relationships between soil chemical properties and soil microbial communities

**Figure 3:**
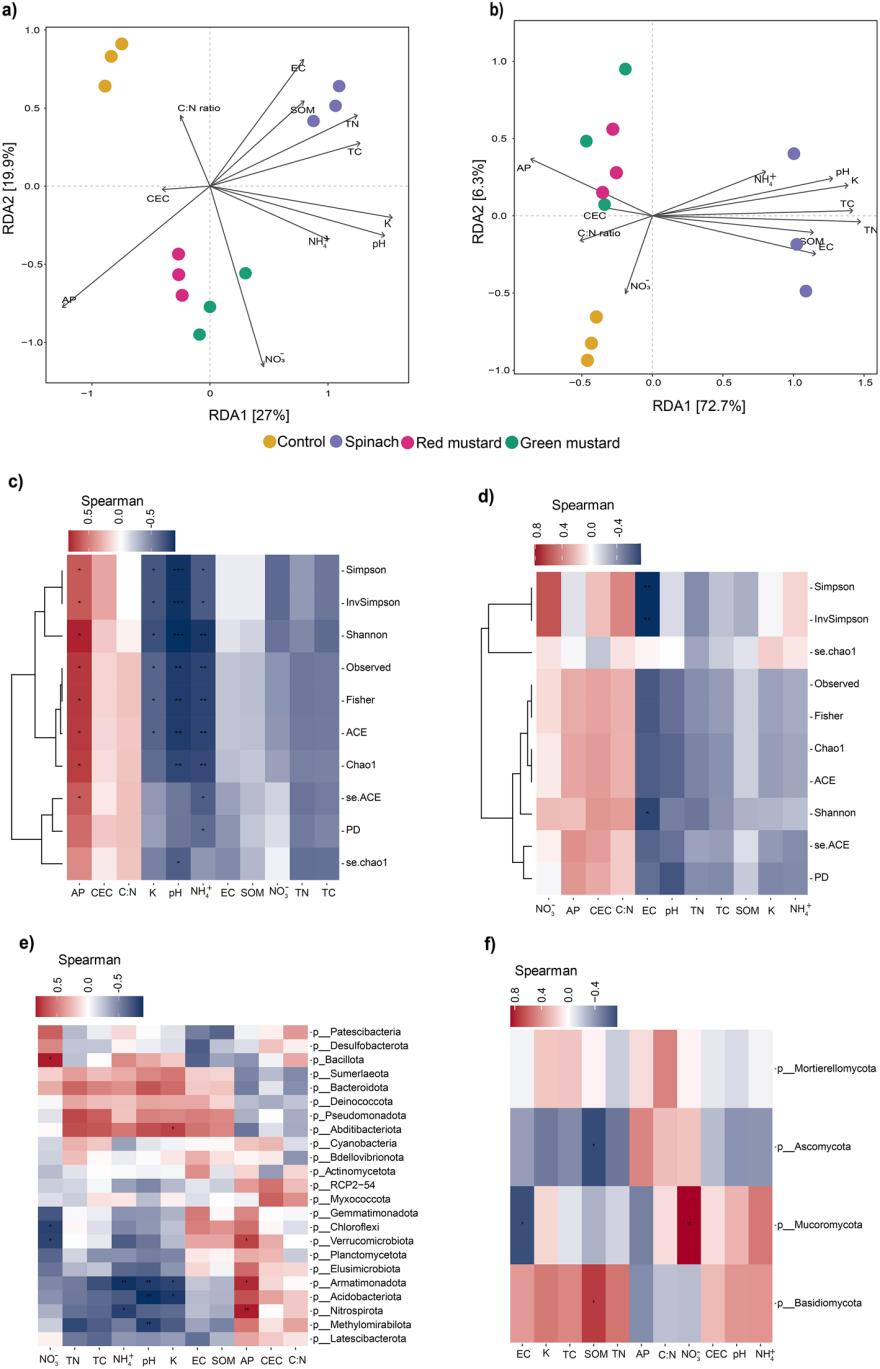
Distance-based redundancy analysis (dbRDA) exhibiting the relationship between soil chemical properties and microbial communities of green manure treatments based on Bray–Curtis distance similarities: bacteria (**a**) and fungi (**b**). The contribution of each soil chemical properties to the soil microbial community structure variation was indicated by the length of arrows. Spearman correlation between soil chemical properties with alpha diversity indices and phylum relative abundance of bacteria (**c**,**e**) and fungi (**d**,**f**). Refer Table 1 for the abbreviation of soil chemical properties.

The impact of soil chemical properties changes following green manuring on microbial community structure (bacteria and fungi) was determined using the Mantel test (Table S3). Soil pH, K, TN, and TC were significantly (*p* ≤ 0.05) correlated with both bacterial and fungal community assemblies (Fig. 3, Table S3). Furthermore, RDA analysis exhibited that the soil chemical properties explained 46.9 and 79.0% of the total bacterial and fungal variation, respectively (Fig. 3a,b). The first two RDA components separated the bacterial and fungal communities in the treatments into three clusters. Bacterial and fungal communities of mustard cultivars-treated soils were clustered together and were separated from control and spinach-treated soils. In the case of bacterial alpha diversity, K, pH and NH_4_^+^ were significantly (*p* ≤ 0.05) negatively correlated with almost all indices of bacterial diversity, whereas soil AP showed a significant positive correlation (Fig. 3c). On the other hand, soil EC was significantly (*p* ≤ 0.05) negatively correlated with fungal diversity (Fig. 3d). Spearman correlation analysis at the phylum level also showed that Acidobacteriota, Nitrospirota and Armatimonadota had a significant (*p* ≤ 0.05) negative correlation with soil pH, NH_4_^+^ and K but a positive correlation to AP (Fig. 3e). Basidiomycota and Ascomycota showed an inverse relationship with soil chemical properties, including the SOM (Fig. 3f).

The SEM analysis also demonstrated that changes in soil chemical properties had a significant (*p* < 0.05) impact on the soil microbiota (Fig. S1). Furthermore, a SEM analysis was performed to determine whether changes in the soil chemical properties affected pepper yield directly or indirectly (through microbiota shift). The results showed that the model was fit and that soil chemical properties, particularly TN and K alteration, had a greater impact pepper fruit yield than soil microbiota (Fig. S1).

### Functional diversity after green manuring

**Figure 4:**
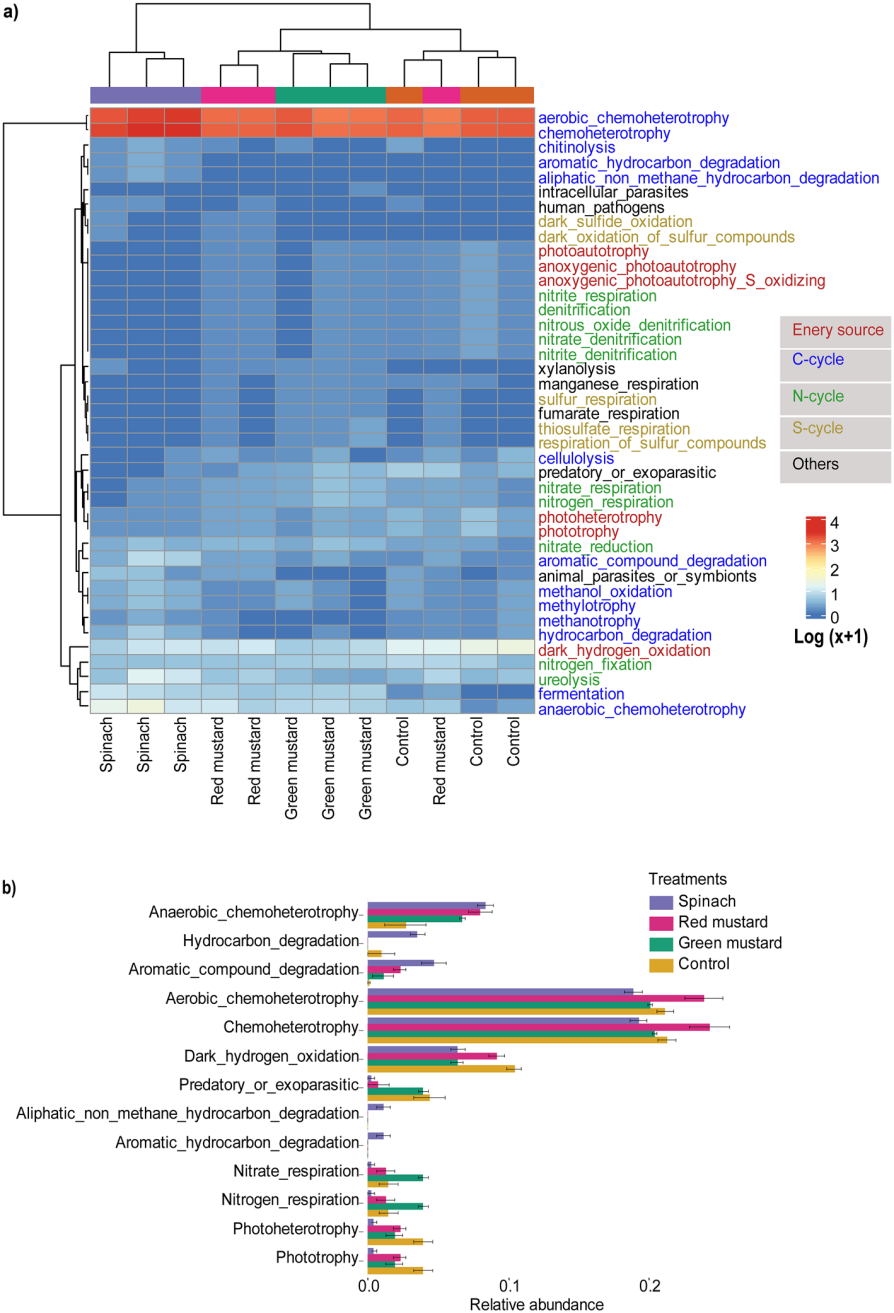
Changes in predicted bacterial functions after green manuring. Heatmap depicting the predicted bacterial ecological functions in different green manures based on the FAPROTAX database (**a**). LEfSe analysis (LDA score > 4.0) of differentially abundant predicted functions among the green manures (**b**).

According to FAPROTAX analysis, a total of 41 predicted bacterial functions were identified from all treatments, with chemoheterotrophy and aerobic chemoheterotrophy being the most functionally redundant predicted functions. Bacterial communities in spinach-amended soil were clustered separately based on their predicted functional profiles (Fig. 4a). LEfSe analysis was performed to identify bacterial functions highly associated with green manures. Among the treatments, spinach-treated soil was differentially abundant in function related to hydrocarbon degradation, while the same treatment had the lowest predicted abundance of nitrate respiration, nitrogen respiration, predatory/exoparasitic, photoheterotrophy, and phototrophy (Fig. 4a). Furthermore, spinach had no predicted denitrification function, whereas green mustard treated-soil showed the highest rates of nitrate and nitrogen respiration. Control, however, showed the lowest abundant functions of anaerobic chemoheterotrophy (Fig. 4a,b). Further correlation analysis showed that anaerobic chemoheterotrophy, fermentation and hydrocarbon degradation were strongly positively associated (*p* ≤ 0.05) with soil pH and K. The soil C:N ratio was positively associated with predicted cellulolysis function. Nitrite and nitrate denitrification showed a significant negative correlation (*p* ≤ 0.05) with K. Soil EC increased the process of functions related to chemoheterotrophy but not with sulfur, fumarate, or manganese respiration (Fig. S2a).

**Figure 5:**
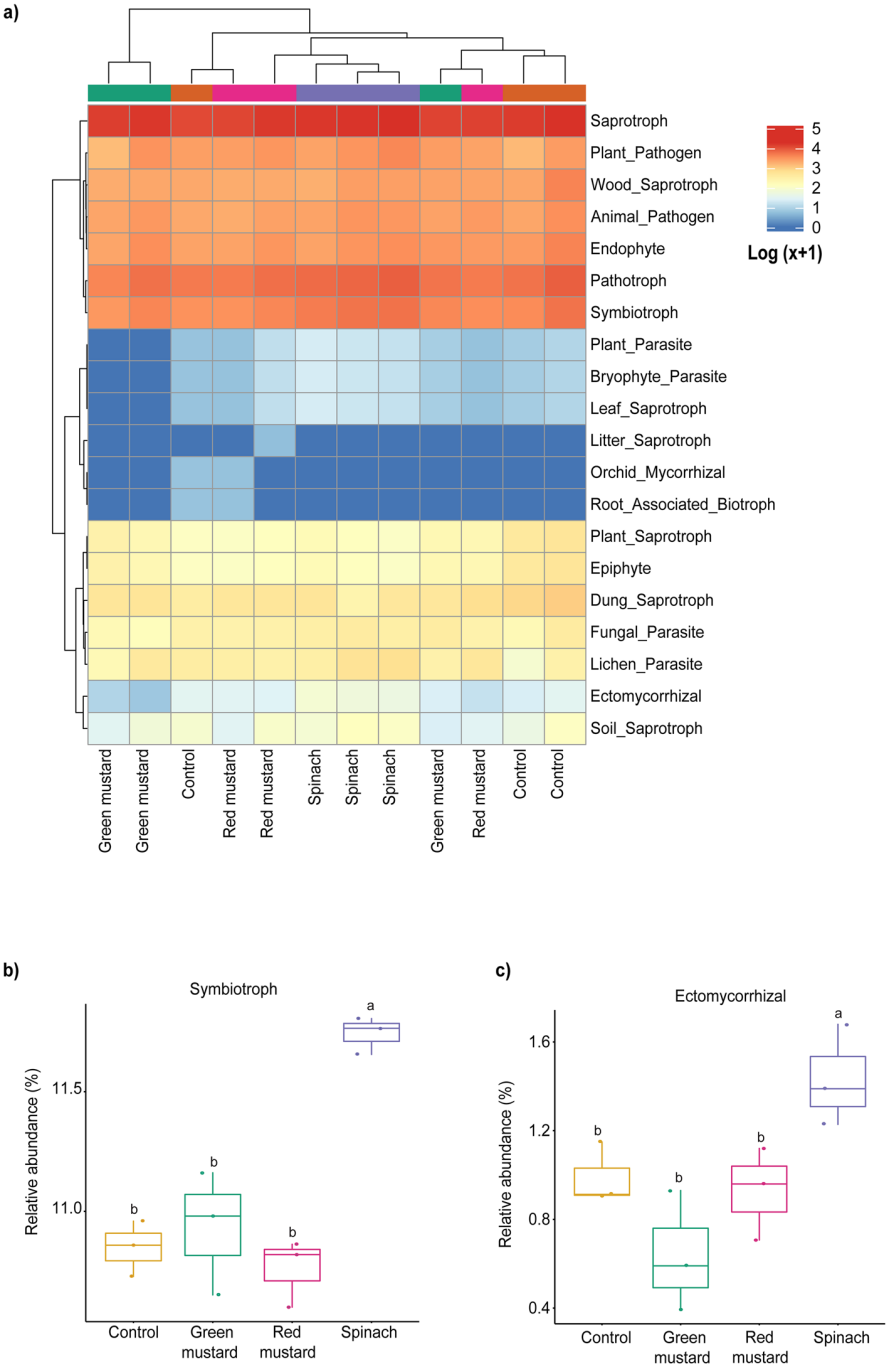
Predicted ecological functions of soil fungal communities in different green manure-amended soils. Heatmap depicting the predicted ecological functions of soil fungal communities in different green manures based on the FUNGuild database (**a**). Spinach-amended soil enriched with symbiotroph (**b**) and ectomycorrhizal (**c**) predicted functions when compared to other green manures and control.

FUNGuild is used to predict changes in the ecological functions of the fungal communities following green manuring. The results showed that the most dominant functional guilds in all treatments were saprotrophs (Fig. 5a). The functional guilds of symbiotrophs and ectomycorrhizal were positively impacted by spinach green manure; however, soil amendments with mustard cultivars did not enhance such predicted functions (Fig. 5b,c). These functions are beneficial to plants because symbiotrophs and ectomycorrhizal fungi make beneficial relationship with plants. Furthermore, some of the predicted functions, including symbiotroph were significantly (*p* > 0.05) associated with soil EC (Fig. S2b).

## Discussion

Soil acidification, soil nutrient depletion, weeds, and soil-borne diseases are major problems in the continuous monocropping of intensive cultivation production systems^28,29^. Thus, alternative solutions are required to tackle these problems^5^. Green manuring is an eco-friendly traditional agricultural practice that improves soil fertility and crop productivity while alleviating impediments to continuing cultivation^1,11,30^. Our study showed that the incorporated spinach green manures resulted in an improvement in soil nutritional conditions (e.g. pH, SOM, TN, NH_4_^+^, and K). Previous reports noted that increased ammonification may contribute to the pH increase in green manure-amended soils^31^. Increased mineralization of organic matter, as seen in spinach with the lowest C:N ratio, enhances the hydroxyl group consumption of H^+^, increasing soil pH^32^. The low pH soil condition might cause high soil AP because of the higher solubility of AP in acidic soil conditions^33^. Thus, addressing the problem of soil acidification would help improve nutrient balance in soil-degraded monocropping agroecosystems^32^. In addition, increased nutrient availability in the green manure-amended soil was observed in our study. Previous studies have revealed that soil nitrogen and exchangeable potassium, following green manuring were the determinant factors for yield enhancement, which is consistent with our work^30^.

Weeds are a major crop production constraint that increases production costs, which necessitates efficient and sustainable weed control. The most effective green manure for suppressing weed populations in our study was spinach followed by mustard. This is consistent with previous reports on weed suppression by various green manures^33^. Even while it is predicted that soil amendment with brassicas will suppress weeds^34^, it was interesting to find that spinach, a non-brassica with no GSL (Table S4), had the highest weed suppression effect. Notably, high fermentation and hydrocarbon degradation (as seen in FAPROTAX predicted function, Fig. 4b) by the presence of abundant *Clostridium* may increase the conversion of carbohydrates to organic acids, which may aid in the suppression of weed growth^35^. Nevertheless, further research is needed to determine why spinach is effective in weed control. Furthermore, spinach had by far the highest fruit yield among green manures, with a yield increase of more than 100% over control. Including spinach in crop rotation increased cucumber yield considerably by increasing beneficial microbes, according to a recent study^26^. The government of South Korea has developed action plans to address challenges with monocropping through sustainable soil management programs^7^. Our study thus raises prospect of encouraging the use of spinach as a green manure preplant soil treatment in the pepper growing regions.

Changes in soil mineral composition following agricultural practices are known to cause a shift in soil microbial community structure and functional diversity^30,36,37^. This can lead to improved crop yield owing to enhanced soil suppressiveness, nutrient cycling, and availability^38,39^ The shift in soil bacterial and fungal community structure after green manure amendment^18,19^ has been previously reported partly because the incorporated substrate modifies the soil nutrient for soil microbe growth and colonization^20^. Our results supports the previous studies that soil pH, K, and TN are major influencing factors in both bacterial and fungal community assemblies^30^. Soil pH, which is associated with cation release during decomposition^40^, is an important factor that markedly influences the bacterial and fungal community structure assembly^41^. Furthermore, the reduced microbial diversity following green manuring can be linked to intra- and inter-kingdom competition caused by changes in soil chemical properties^30,42,43^. In our study, where green manures altered soil nutritional status, bacterial diversity was significantly negatively associated with NH_4_^+^. Previous research has also found that soil bacterial diversity is highly negatively associated with N application^23^. Green manuring with low GSL-containing red mustard cultivar had higher microbial diversity than green mustard cultivar with high GSL content. Although GSL is known to have negative effect on microbial diversity, more research is required to confirm it^34^.

Our results support previous reports that many members of Bacillota, including *Clostridium, Bacillus* and *Sedimentibacter,* were enriched in response to green manure soil amendments. Bacillota are copiotrophs in which substrate-amendment enhances nutrient-rich environment for their growth^4,21,22^. The genus *Clostridium* are diazotrophs capable nitrogen fixation^10,44^ and the production of toxic organic acids that could suppress soil-borne pathogens and weeds^29,45,46^. On the other hand, Chloroflexi and Acidobacteriota were reduced with green manure addition. These findings comply with the previous studies that such oligotrophic bacteria are adapt to low available soil nutrients^23^ and low pH conditions^3,20,47^. Chloroflexi are often strongly associated with low crop productivity^3,48^, and some studies label them as disease inducible^49^. In addition, the majority of Chloroflexi members do not fix nitrogen; instead, they compete with other beneficial microbes and the host plant itself for nitrogen resources^4,50^. Basidiomycota and Ascomycota had an inverse relationship with soil chemical properties as reported in the previous study^51^. The positive correlation between Basidiomycota and soil organic matter supports the previous report that Basidiomycota are the primary decomposers of soil debris^51^. *Fusarium* was differentially more abundant in control as opposed to the spinach-amended soil. *Fusarium* is serious soil-borne pathogens that affect a variety of crops, including peppers, and are well adapted to pepper monoculture^24,52^. The reduction in *Fusarium* abundance with organic amendment complies with the previous finding^24^, suggesting the potential of spinach as green manure for the suppression of *Fusarium*-incited diseases. Furthermore, the enrichments of beneficial soil fungi, such as *Papiliotrema*^53,54^, that have the potential to engage in biocontrol activities following spinach amendment shows that spinach as green manure not only improves soil nutrition but also promotes resident soil microbes with biocontrol potential to flourish.

In summary, spinach improved soil nutrition (e.g., pH, SOM, TN, NH_4_^+^, and K), pepper growth, pepper fruit yield and suppressed weed population. Green mustard also increased soil nutrition and suppressed weed growth but had no significant effect on pepper yield. The major influencing factors in both bacterial and fungal community assemblies were soil pH, TC, TN, and K. All green manures highly stimulated members of Bacillota, including *Clostridium* and *Bacillus*. Spinach also highly reduced the abundance of members of Acidobacteriota and Chloroflexi while enriching fungal members of Rhynchogastremataceae, such as *Papiliotrema*. Overall, spinach outperformed other treatments in terms of weed control and yield improvement, whereas red mustard exceeded for positive effect on soil fungal diversity. This study contributes significantly to our understanding of how the soil microbiome and soil fertility alteration via green manure application as a pre-plant soil treatment might help alleviate continuous cropping obstacles.

## Materials and methods

### Materials, study design and sampling

Seeds of mustard cultivars (green and red mustard) were acquired from the National Institute of Crop Science (NICS), Rural Development Administration, South Korea. Spinach seeds were obtained from Jeilseed Company in Doan-myeon, Chungcheongbuk-do, South Korea. Seeds of these *Brassica* cultivars and spinach were planted in a polyhouse at Kyungpook National University, South Korea, and plant biomass was collected two months after planting. The soil for this study pot experiment was collected in January 2021 from a long-year pepper-monocropped soil in Gunwi-gun, Gyeongsangbuk-do province, South Korea (36°10′09′′N,128°38′24′′E), whose productivity had declined substantially (Fig. S3). The soil was sieved through an 8-mm sieve and completely homogenized. The initial soil chemical properties are indicated in the Table S5.

The fresh harvested biomass of green manures, which contained a variable range of total GSL concentrations (Table S4), was mix homogeneously and separately with the soil at 0.5% (w/w) on a dry weight basis. Soil with no green manure amendment served as the non-amended control. The soil from each treatment group was placed in plastic containers with three replicates. Each treatment, including control, was watered (sterile distilled water) to 70% field capacity and covered for 30 days (Start date: June 6, 2021 End date: February 5, 2021) with a plastic transparent polythene film in the polyhouse. The polythene film was uncovered and the soil was air-drained for 60 days (Start date: February 5, 2021 End date: April 9, 2021). Pots (15 cm diameter, 31 cm height, aerated with holes at the bottom) were filled with 2 kg green manure-amended soil (10 g pot ^−1^). One pepper (cultivar Dongmudae) seedling, one-month-old, was transplanted into each pot (April 9, 2021). Four different treatments were used in the current study: control, spinach, red mustard and green mustard. All treatments were replicated three times and laid out in a completely randomized experimental design, with each replicate containing five pots (15 pots per treatment). Pepper plants were grown in a polyhouse for three months (Start date: April 2021 End date: July 2021) and watered twice a week. Soil samples for chemical property analysis and DNA extraction were collected after soil treatment immediately before transplantation. Soil samples were collected at three points within each pot and the samples were pooled to yield three pooled samples (replicates). The soil samples were kept at − 80 °C until the DNA extraction.

### Soil chemical analysis

The soil chemical properties were analyzed from dried soil samples. Using a pH and EC meter (SP2000, Skalar BV, Netherlands), the electrical conductivity (EC) and pH of the soil were determined in a 1:5 (w/v) soil: deionized distilled water suspension. A titrando automatic titrator (Metrohm 888, Switzerland) was used to analyze soil organic matter (SOM). The BaCl_2_-H_2_SO_4_ exchange method^55^ was used to determine the soil cation exchange capacity (CEC). The ammonium-nitrogen (NH_4_^+^) nitrate-nitrogen (NO_3_^−^) concentration in the soil were measured colorimetrically by salicylate method^56^ and cadmium reduction method^57^, respectively, using BLTEC QuAAtro (BLTEC KK, Japan). The soil total nitrogen (TN) concentration was determined by the method described by Dumas^58^ with S832DR (Leco, USA). Soil exchangeable potassium (K) concentration was analyzed using a PerkinElmer® Optima 8300 ICP-OES (PerkinElmer, Inc., MA, USA). The soil available P_2_O_5_ (AP) concentration was analyzed using a SKALAR San +  + system autoanalyzer (Skalar Analytical B.V., Breda, Netherlands).

### Weed emergence and pepper performance

The reduction in the emergence counts of monocot and dicot weeds following green manure application was determined before pepper transplanting. Pepper growth parameters, such as plant height, stem diameter, primary branch length and diameter, and chlorophyll content, were measured at the end of the experiment (July 2021), three months after transplanting. Chlorophyll content (SPAD unit) was measured using a chlorophyll-meter (Konica Minolta, Japan). Fully shiny matured green fruits with over 5 cm were collected three times.

### DNA extraction, library preparation and sequencing

The DNeasy® PowerSoil® Pro Kit (Qiagen, Hilden, Germany) was to used extract microbial DNA from soil samples (0.5 g) according to the manufacturer’s protocol. The extracted DNA quantity and purity were measured using a Qubit® 2.0 Fluorometer (Thermo Fisher Scientific, Waltham, MA, USA) and NanoDrop™ One^C^ spectrophotometer (Thermo Fisher Scientific). The extracted DNA was stored at − 80 °C until it was used for Illumina MiSeq sequencing.

The fungal internal transcribed spacer 1 (ITS1) region and the bacterial V4-V5 hypervariable region of the 16S rRNA gene were PCR-amplified with ITS86F/ITS4R^59,60^ and the universal primers 515F/907R^61^, using an Eppendorf Mastercycler® Nexus PCR Cycler (Eppendorf, Hamburg, Germany). The 50 µl PCR reaction mixture included 25 µl EmeraldAmp® PCR Master Mix (Takara, Shiga, Japan), 1 µl DNA template, 1 µl (0.5 µM/µl) per primer, and 22 µl of double-distilled water. The PCR reaction conditions and primer sequences are shown in Table S6. The Nextera®XT Index Kit (Illumina, San Diego, CA, USA) was used to ligate the Illumina sequence adapters to the PCR products, according to the manufacturer’s protocol. The final PCR products were purified using AMPure XP beads (Beckman Coulter Life Sciences, CA, USA) and kept at − 20 °C until use. The size variation in the amplicon product was considered while pooling samples of both 16S rRNA and ITS2 indexed amplicons at equimolar concentration. The libraries were checked for their concentration and size using an Agilent Bioanalyzer (Santa Clara, Ca, USA), and the pooled library with a final loading concentration of 20 pM was sequenced using the Illumina MiSeq platform (Illumina) at Kyungpook National University’s NGS Core Facility Center in South Korea.

### Bioinformatics analysis

Bacterial and fungal raw sequences were demultiplexed using the QIIME2 pipeline (https://qiime2.org), and the reads were denoised in QIIME2 using DADA2, and chimeric sequences and singletons were removed^62^. Reads were truncated, and the ones with quality scores of ≥ 25 were retained. Non-chimeric representative sequences that made up amplicon sequence variants (ASVs) were aligned using MAFFT^63^ and taxonomy was assigned using a classify-sklearn-based qiime feature-classifier trained on the reference SILVA 99% full-length database (version 138.1)^64^ and UNITE database (version 8.3)^65^ for bacteria and fungi, respectively. ASVs assigned as mitochondria, chloroplasts, and unclassified taxa at the kingdom level were excluded. The sample reads were rarefied to equal size to enable a similarity comparison between treatments. The normalized data set contained 1786 and 202 ASVs of bacteria and fungi, respectively. FAPROTAX, functional annotation of prokaryotic taxa, was used to predict the ecological functions of bacterial communities^66–68^. Fungal functional guild (FUNGuild)^69^ was used to predict the functional changes in fungal communities following BF treatment.

### Statistical analysis

All downstream statistical data analyses were conducted using the R statistical software (v4.1.3)^70^. Data visualization was performed using different R packages: ggplot^71^ and ComplexHeatmap (neatmap v2.1.0)^72^. Homogeneity of variance and multivariate homogeneity of dispersion were checked using Levene’s test and PERMDISP^73,74^, respectively. The data normality assumption was tested using the Shapiro–Wilk test. ANOVA with Duncan’s multiple range test with dplyr package were used to compare the statistical difference between treatments in soil chemical properties, plant phenotype and alpha diversity indices (at ASVs-level). The overall statistical difference in microbial community composition between treatments was determined using permutational multivariate analysis of variance (PERMANOVA) (Adonis; vegan, version 2.5.7)^75^. The association between soil chemical properties and abundance of soil microbial communities was assessed using dbRDA in R. LEfSe^76^, metastat^77^, metagenomeSeq^78^, and Random forest^79^ in R were used to identify potential microbial biomarkers that were statistically differentially abundant between control and green manure-amended treatments.

Using the vegan and sem packages in R (v4.1.3)^70^, structural equation modeling (SEM) analysis was carried out to comprehend how a change in the soil chemical properties and microbial community following the addition of green manure effects pepper yield. Additionally, the of first PCOA values were served as a representation of the bacterial and fungal community structures in the SEM analysis^80^. Microbial diversity was a representation of bacterial and fungal diversities. Low chi-square (X2) value/degree of freedom (< 2), non-significant X2 test (*p* > 0.05), a low root mean squared error of approximation (RMSEA < 0.05), high comparative fit index (CFI > 0.9) and low standard root mean square residual (SRMR < 0.05) were used to determine the model’s fit.

## Supplementary Information

Supplementary Information 1.

Supplementary Information 2.

## Data Availability

Under the PRJNA857858 BioProject, all raw sequences of bacteria and fungi are available at the NCBI Sequence Read Archive (SRA) repository (SRX Accessions SRX16121282-SRX16121301).

## Research involving plants

The use of plants in our experimental research complies with all local, national, and international guidelines and legislations.
