# Supplementary material for: Spinach (Spinacia oleracea) as green manure modifies the soil nutrients and microbiota structure for enhanced pepper productivity
Source: Sci Rep. 2023 Mar 13;13:4140. doi: 10.1038/s41598-023-31204-8 (PMC10011398; doi:10.1038/s41598-023-31204-8)
Supplement: Supplementary file 1 — Supplementary Information 1. [file 41598_2023_31204_MOESM1_ESM.docx]

**Spinach (*Spinacia oleracea*) as Green Manure Modifies the Soil Nutrients and Microbiota Structure for Enhanced Pepper Productivity**

Ryeong-Hui Kim^1, +^, Setu Bazie Tagele^2,3, +^, Minsoo Jeong^2^, Da-Ryung Jung^2^, Dokyung Lee^1^, TaeHyung Park^1^, Bashizi Flory Tino^2^, Kyeongmo Lim^2^, Min A Kim^2^, Yeong-Jun Park^3^, and Jae-Ho Shin^1,2,3, *^

^1^*Department of Integrative Biology, Kyungpook National University, Daegu 41566, Republic of Korea*

^2^*Department of Applied Biosciences, Kyungpook National University, Daegu 41566, Republic of Korea*

^3^*NGS core facility, Kyungpook National University, Daegu 41566, Republic of Korea*

*Correspondence: [jhshin@knu.ac.kr](mailto:jhshin@knu.ac.kr) (J.H.S); Tel.: +82-53-950-5716; Fax: +82-53-953-7233

^+^these authors contributed equally to this work

**Supplementary**

**Table S1.** Alteration of alpha diversity indices of bacterial and fungal diversity indices following green manuring

|  | Green manures | | | |
| --- | --- | --- | --- | --- |
|  | Control | Spinach | Red mustard | Green mustard |
| **Bacteria** |  |  |  |  |
| Observed | 428 ± 1^a^ | 340.4 ± 4.71^c^ | 417 ± 14.85^ab^ | 384.7 ± 19.35^b^ |
| Chao1 | 430.9 ± 1.82^a^ | 340.5 ± 4.68^c^ | 419.8 ± 15.22^ab^ | 385.7 ± 19.91^b^ |
| ACE | 429.4 ± 1.03^a^ | 340.7 ± 4.65^c^ | 418.8 ± 14.95^ab^ | 385.4 ± 19.5^b^ |
| Shannon | 5.8 ± 0.01^a^ | 5.5 ± 0.06^c^ | 5.7 ± 0.03^ab^ | 5.6 ± 0.06^bc^ |
| Simpson | 0.996 ± 0.01^a^ | 0.993 ± 0.01^b^ | 0.995 ± 0.01^ab^ | 0.994 ± 0.01^b^ |
| InvSimpson | 262.9 ± 2.71^a^ | 166.8 ± 19.75^c^ | 205.4 ± 1.45^b^ | 187.9 ± 9.85^bc^ |
| Fisher | 93.6 ± 0.28^a^ | 70 ± 1.22^c^ | 90.6 ± 4.09^ab^ | 81.8 ± 5.22^b^ |
| Coverage | 1 ± 0.01^a^ | 1 ± 0.01^a^ | 1 ± 0.01^a^ | 1 ± 0.01^a^ |
| PD | 114 ± 1.72^a^ | 94.2 ± 1.67^b^ | 119.4 ± 4.14^a^ | 111.1 ± 3.85^a^ |
| **Fungi** |  |  |  |  |
| Observed | 71 ± 8.72^b^ | 40.7 ± 2.41^c^ | 89.4 ± 2.34^a^ | 68 ± 4.94^b^ |
| Chao1 | 71.2 ± 8.82^b^ | 41.2 ± 2.48^c^ | 89.7 ± 2.19^a^ | 68.2 ± 4.93^b^ |
| ACE | 71.7 ± 8.99^b^ | 41.5 ± 2.44^c^ | 89.6 ± 2.23^a^ | 68.4 ± 4.82^b^ |
| Shannon | 2.5 ± 0.09^b^ | 2 ± 0.21^c^ | 3.1 ± 0.08^a^ | 2.6 ± 0.16^ab^ |
| Simpson | 0.81 ± 0.02^ab^ | 0.74 ± 0.06^b^ | 0.89 ± 0.01^a^ | 0.85 ± 0.02^a^ |
| InvSimpson | 5.3 ± 0.3^bc^ | 4.4 ± 1.02^c^ | 8.8 ± 0.42^a^ | 6.9 ± 0.64^ab^ |
| Fisher | 11.4 ± 1.66^b^ | 5.9 ± 0.41^c^ | 14.9 ± 0.47^a^ | 10.8 ± 0.93^b^ |
| Coverage | 0.99 ± 0.01^a^ | 0.99 ± 0.01^a^ | 0.99 ± 0.01^a^ | 0.99 ± 0.01^a^ |
| PD | 11.7 ± 1.6^a^ | 7.7 ± 0.47^b^ | 13.7 ± 0.39^a^ | 10.6 ± 0.84^ab^ |

Mean values followed by different letter (s) within a row (of alpha diversity index) represent significant differences at P < 0.05, DMRT test.

**Table S2.** Composition of bacterial and fungal communities at phylum level following green manuring

|  | Green manures | | | |
| --- | --- | --- | --- | --- |
|  | Control | Spinach | Red mustard | Green mustard |
| **Bacteria** |  |  |  |  |
| Pseudomonadota | 27.6 ± 0.03^b^ | 33.8 ± 0.02^a^ | 25.3 ± 0.01^b^ | 27.5 ± 0.02^b^ |
| Bacillota | 8.4 ± 0.02^c^ | 24.3 ± 0.01^b^ | 30.8 ± 0.02^a^ | 31.8 ± 0.03^a^ |
| Acidobacteriota | 17.2 ± 0.02^a^ | 3.6 ± 0.02^c^ | 11.6 ± 0.01^b^ | 8.3 ± 0.01^b^ |
| Chloroflexi | 13.4 ± 0.02^a^ | 8.1 ± 0.01^b^ | 8 ± 0.01^b^ | 6.4 ± 0.01^b^ |
| Actinomycetota | 11.5 ± 0.03^a^ | 9.3 ± 0.02^ab^ | 5.7 ± 0.01^b^ | 6.4 ± 0.01^ab^ |
| Bacteroidota | 3.6 ± 0.02^c^ | 7.7 ± 0.01^a^ | 4.5 ± 0.01^bc^ | 7.1 ± 0.01^ab^ |
| Planctomycetota | 5.1 ± 0.01^a^ | 4.1 ± 0.01^a^ | 4.6 ± 0.01^a^ | 3.8 ± 0.01^a^ |
| Gemmatimonadota | 5.1 ± 0.01^a^ | 4.2 ± 0.01^a^ | 2.5 ± 0.01^b^ | 3 ± 0.01^b^ |
| Verrucomicrobiota | 3.7 ± 0.01^a^ | 2 ± 0.01^b^ | 2.4 ± 0.01^b^ | 1.7 ± 0.01^b^ |
| Myxococcota | 1.9 ± 0.01^a^ | 1.8 ± 0.01^a^ | 2.2 ± 0.01^a^ | 1.7 ± 0.01^a^ |
| Desulfobacterota | 0.3 ± 0.01^ab^ | 0.2 ± 0.01^b^ | 0.8 ± 0.01^a^ | 0.8 ± 0.01^a^ |
| Armatimonadota | 0.9 ± 0.01^a^ | 0.2 ± 0.01^c^ | 0.6 ± 0.01^b^ | 0.4 ± 0.01^bc^ |
| RCP2-54 | 0.5 ± 0.01^ab^ | 0.3 ± 0.01^b^ | 0.4 ± 0.01^a^ | 0.4 ± 0.01^a^ |
| Nitrospirota | 0.4 ± 0.01^a^ | 0.2 ± 0.01^b^ | 0.4 ± 0.01^a^ | 0.4 ± 0.01^a^ |
| Cyanobacteria | 0.4 ± 0.01^a^ | 0.3 ± 0.01^a^ | 0.3 ± 0.01^a^ | 0.4 ± 0.01^a^ |
| Others | 0.8 ± 0.01^a^ | 0.6 ± 0.01^a^ | 0.7 ± 0.01^a^ | 0.8 ± 0.01^a^ |
| **Fungi** |  |  |  |  |
| Ascomycota | 96.6 ± 0.86^ab^ | 90.06 ± 3.99^ab^ | 95.04 ± 1.4^a^ | 98.76 ± 0.33^a^ |
| Basidiomycota | 3.3 ± 0.89^a^ | 9.9 ± 3.97^a^ | 4.54 ± 1.28^a^ | 1.09 ± 0.33^a^ |
| Mucoromycota | 0 ± 0^a^ | 0.02 ± 0.01^a^ | 0.12 ± 0.08^a^ | 0.08 ± 0.01^a^ |
| Mortierellomycota | 0 ± 0^a^ | 0 ± 0^a^ | 0.01 ± 0.01^a^ | 0 ± 0^a^ |
| Others | 0.1 ± 0.08^a^ | 0.02 ± 0.01^a^ | 0.29 ± 0.16^a^ | 0.08 ± 0^a^ |

Mean values followed by different letter (s) within a row (phylum) represent significant differences at p ≤ 0.05, DMRT test.

**Table S3**. The Pearson’s correlation (r) between microbial community structure based on Bray−Curtis distance and soil chemical properties as determined by the Mantel test

| Soil chemical properties | Bacteria | |  | Fungi | |
| --- | --- | --- | --- | --- | --- |
|  | Correlation coefficient | Adjusted p value |  | Correlation coefficient | Adjusted p value |
| pH | 0.77 | 0.004 |  | 0.544 | 0.019 |
| AP | 0.643 | 0.004 |  | 0.039 | 0.302 |
| K | 0.856 | 0.004 |  | 0.704 | 0.004 |
| NO_3_^-^ | 0.385 | 0.020 |  | -0.026 | 0.601 |
| NH_4_^+^ | 0.377 | 0.015 |  | 0.086 | 0.358 |
| TC | 0.409 | 0.013 |  | 0.745 | 0.004 |
| TN | 0.45 | 0.011 |  | 0.824 | 0.004 |
| C:N ratio | -0.139 | 0.862 |  | -0.091 | 0.666 |
| EC | 0.239 | 0.065 |  | 0.486 | 0.035 |
| SOM | 0.148 | 0.133 |  | 0.486 | 0.02 |
| CEC | -0.079 | 0.790 |  | -0.121 | 0.696 |

Exchangeable potassium (K), total nitrogen (TN), ammonium nitrogen (NH_4_^+^), available P_2_O_5_ (AP), soil organic matter (SOM), nitrate nitrogen (NO_3_^-^), electrical conductivity (EC), cation exchange capacity (CEC).

**Table S4.** Total nutrient content and total glucosinolates content of green manures

| Green manures | Scientific name | Cultivar name | Total N | | Total P | | Total K | | Total glucosinolates (μmol g^-1^) * |
| --- | --- | --- | --- | --- | --- | --- | --- | --- | --- |
|  |  |  | Concentration (g kg^-1^) | Added nutrient (g pot^-1^) | Concentration (g kg^-1^) | Added nutrient (g pot^-1^) | Concentration (g kg^-1^) | Added nutrient (g pot^-1^) |  |
| Spinach | *Spinacia oleracea* | Namdongcho | 56.5 | 0.565 | 5.6 | 0.056 | 74.5 | 0.745 | N/A |
| Red mustard | *Brassica juncea* | Jeil Red | 47.4 | 0.474 | 6.8 | 0.068 | 53 | 0.053 | 10.83 |
| Green mustard | *Brassica juncea* | Jeil Cheong | 63.4 | 0.634 | 5.6 | 0.056 | 58.3 | 0.583 | 20.85 |

^*^N/A=not detected

**Table S5.** Soil chemical properties of the pre-treatment soil

| **Soil chemical properties** | **Concentration** |
| --- | --- |
| pH | 5.8 |
| EC ^(^dS m^-1^) | 1.12 |
| CEC (cmol_c_ kg^-1^) | 18.18 |
| SOM ^(^g kg^-1^) | 31.89 |
| TC (g kg^-1^) | 18.5 |
| TN (g kg^-1^) | 2.0 |
| C:N ratio | 9.25 |
| NO_3_^-^ (mg kg^-1^) | 82.7 |
| NH_4_^+^ (mg kg^-1^) | 5.2 |
| AP (mg kg^-1^) | 679.67 |
| K (cmol_c_ kg^-1^) | 0.65 |

Electrical conductivity (EC), cation exchange capacity (CEC), soil organic matter (SOM), total nitrogen (TN), nitrate nitrogen (NO_3_^-^), ammonium nitrogen (NH_4_^+^), available P_2_O_5_ (AP), Exchangeable potassium (K).

**Table S6.** Sets of primers used in this study and the PCR conditions used in Illumina sequencing

| Target gene | Primer set | Sequences (5’-3’) | PCR reaction conditions |
| --- | --- | --- | --- |
| Bacterial V4-V5 region of the 16S rRNA gene | 515F | GTGCCAGCMGCCGCGGTAA | Initial denaturation at 95℃ for 3; followed by 20 cycles at 95℃for 30 s, at 56℃for 30 s, at 72℃for 30 s; final elongation at 72℃ for 5 min. |
|  | 907R | CCGYCAATTCMTTTRAGTTT |  |
| Fungal ITS region | ITS86F | GTGAATCATCGAATCTTTGAA | Initial denaturation at 95℃ for 5; followed by 30 cycles at 95℃for 30 s, at 58℃for 30 s, at 72℃for 30 s; final elongation at 72℃ for 5 min. |
|  | ITS4R | TCCTCCGCTTATTGATATGC |  |

# ***Supplementary figures***


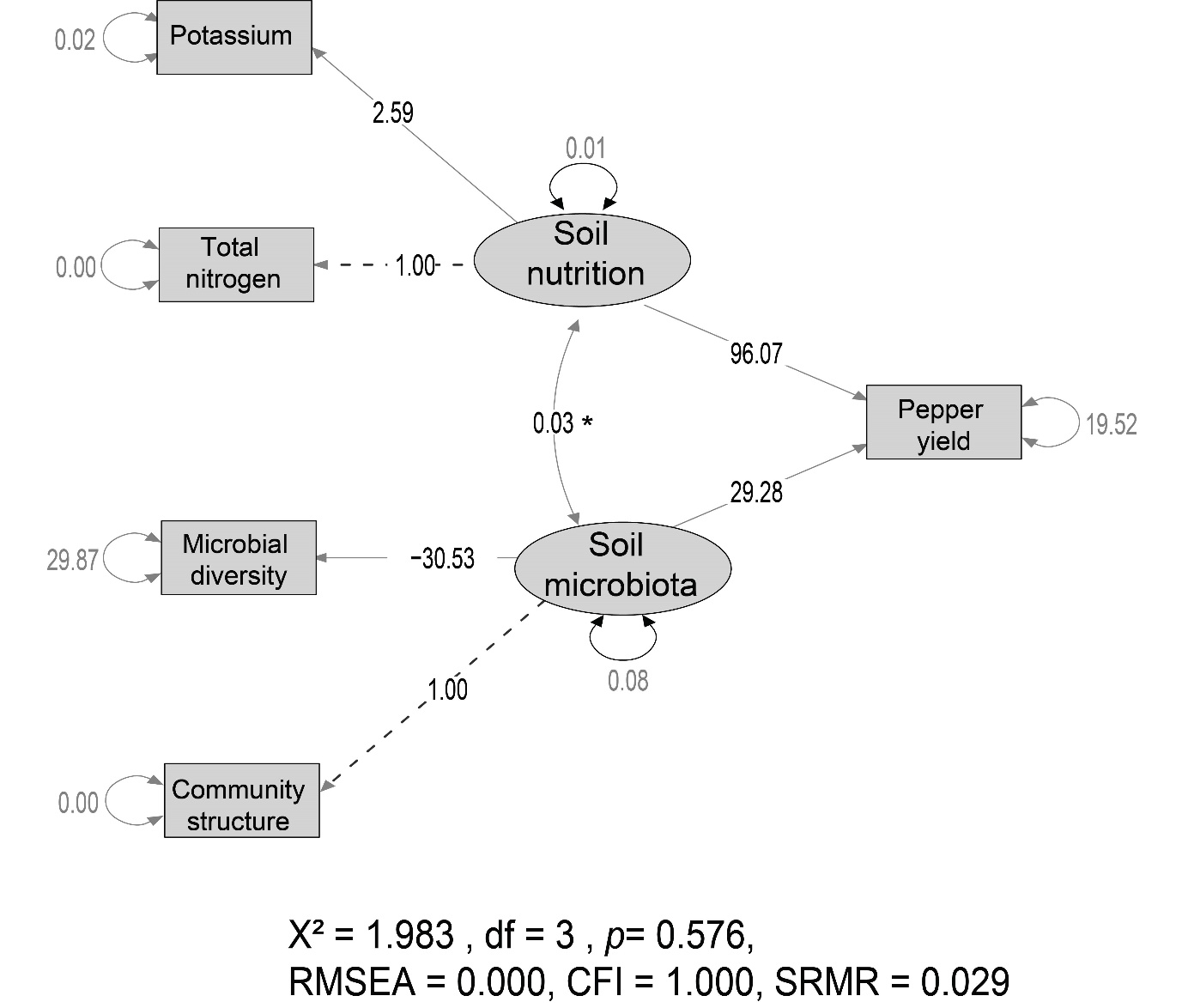
Fig. S1: **The structural equation modelling (SEM) depicts the impact of the soil chemical properties and microbial community changes on pepper yield following the addition of green manure.** Observed variables are shown as rectangles and latent variables as ovals. The dashed lines represent bench mark fixed values. A high loading factor indicates that the observed variable for each latent variable is strongly represented. Negative values indicate the negative relationship between the manifest variable and the constructed latent variable. Double headed arrows represent the variances and residuals for each variable.

**
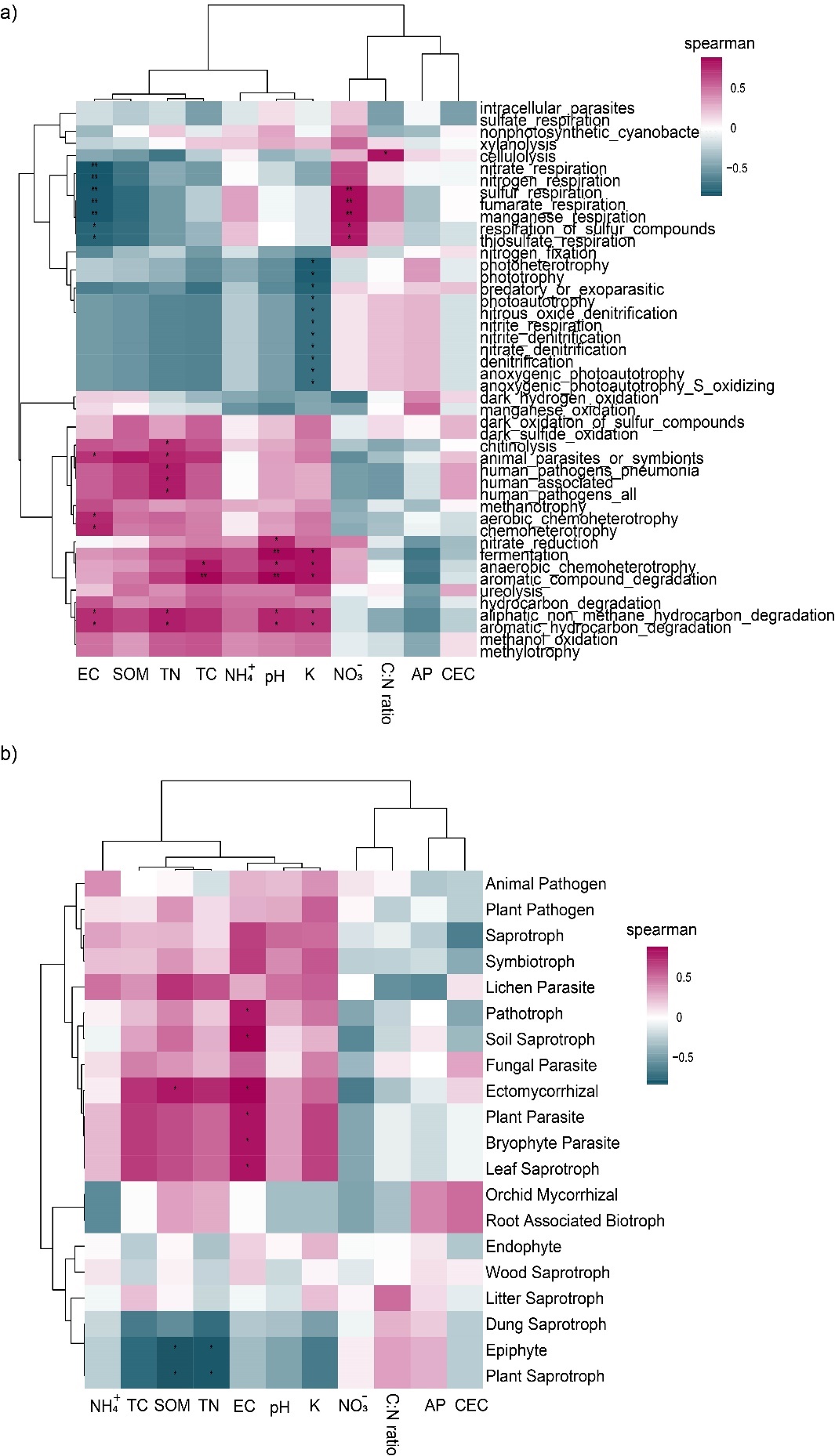
**

**Figure S2. Heat map showing the Spearman’s rank correlation analysis of soil chemical properties with predicted soil ecological functions.** FAPROTAX (**a**) and FUNGuild (**b**) predicted functions. * p<0.05, **p<0.01, ***p<0.001. Refer Table S5 for the abbreviation of soil chemical properties.

**
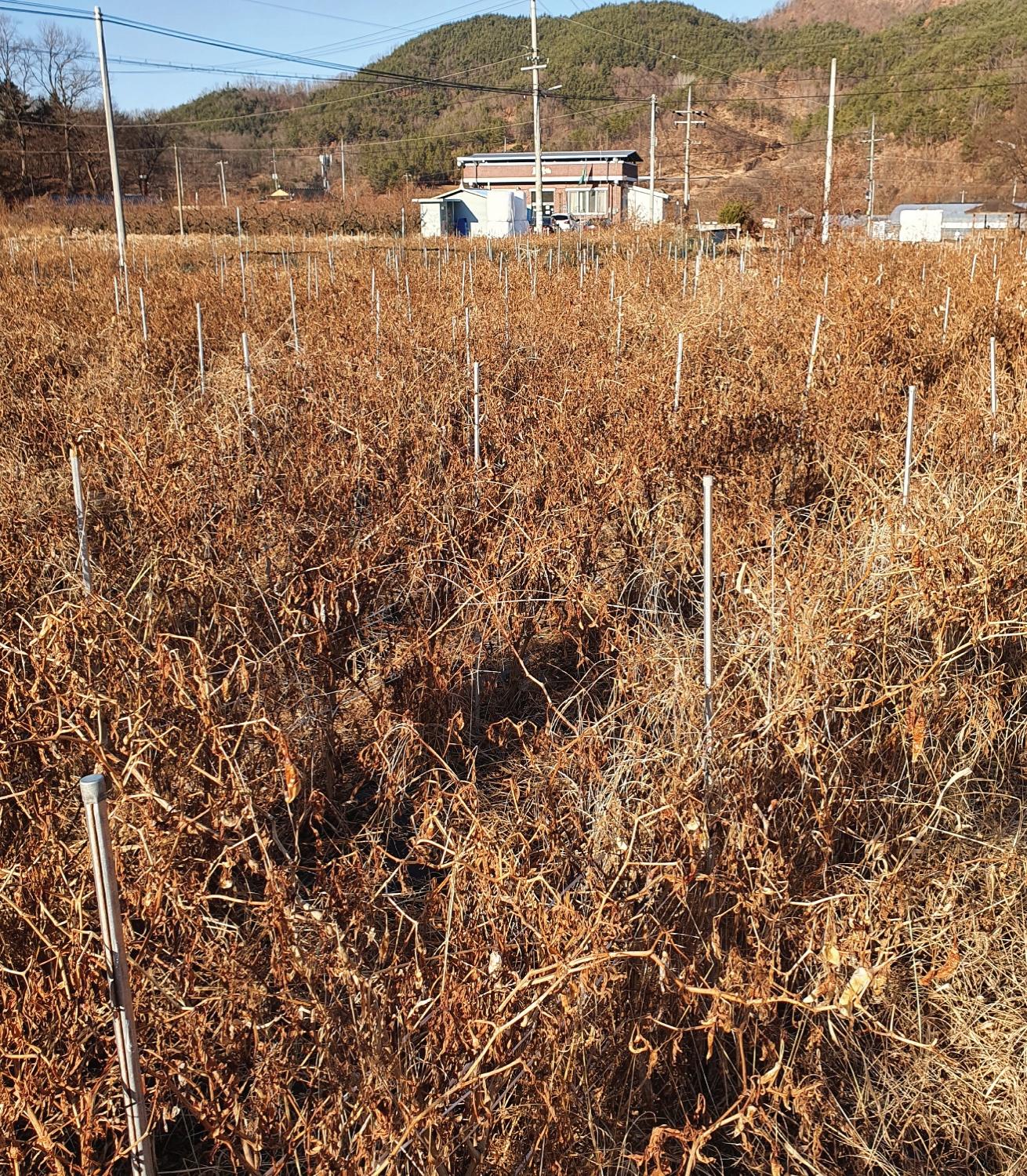
**

**Fig. S3. Land scape of the soil sampled field.**
